# Prostate MRI Using Deep Learning Reconstruction in Response to Cancer Screening Demands—A Systematic Review and Meta-Analysis

**DOI:** 10.3390/jpm15070284

**Published:** 2025-07-02

**Authors:** Stephan Ursprung, Georgios Agrotis, Petra J. van Houdt, Leon C. ter Beek, Thierry N. Boellaard, Regina G. H. Beets-Tan, Derya Yakar, Anwar R. Padhani, Ivo G. Schoots

**Affiliations:** 1 Department of Radiology, The Netherlands Cancer Institute, 1066 CX Amsterdam, The Netherlands; stephan.ursprung@med.uni-tuebingen.de (S.U.);; 2 Department of Diagnostic and Interventional Radiology, Tübingen University Hospital, Karls-Eberhardt University, 72076 Tübingen, Germany; 3 GROW—Innovative Cancer Diagnostics & Therapy, Faculty of Health, Medicine and Life Sciences, University of Maastricht, 6229 ER Maastricht, The Netherlands; 4 Department of Radiation Oncology, The Netherlands Cancer Institute, 1066 CX Amsterdam, The Netherlands; 5 Department of Medical Physics, The Netherlands Cancer Institute, 1066 CX Amsterdam, The Netherlands; 6 Department of Radiology, University Medical Center Groningen, 9713 GZ Groningen, The Netherlands; 7 Paul Strickland Scanner Centre, Mount Vernon Cancer Centre, Northwood HA6 2RN, UK; 8 Department of Radiology and Nuclear Medicine, Erasmus MC Cancer Institute, University Medical Centre, 3015 CP Rotterdam, The Netherlands

**Keywords:** prostate cancer, MRI, deep learning reconstruction, acceleration, systematic review

## Abstract

**Background/Objectives**: There is a growing need for efficient prostate MRI protocols due to their increasing use in managing prostate cancer (PCa) and potential inclusion in screening. Deep learning reconstruction (DLR) may enhance MR acquisitions and improve image quality compared to conventional acceleration techniques. This systematic review examines DLR approaches to prostate MRI. **Methods**: A search of PubMed, Web of Science, and Google Scholar identified eligible studies comparing DLR to conventional reconstruction for prostate imaging. A narrative synthesis was performed to summarize the impact of DLR on acquisition time, image quality, and diagnostic performance. **Results**: Thirty-three studies showed that DLR can reduce acquisition times for T_2_w and DWI imaging while maintaining or improving image quality. It did not significantly affect clinical tasks, such as biopsy decisions, and performed comparably to human readers in PI-RADS scoring and the detection of extraprostatic extension. However, AI models trained on conventional data might be less accurate with DLR images. The heterogeneity in image quality metrics among the studies prevented quantitative synthesis. **Discussion**: DLR has the potential to achieve substantial time savings in prostate MRI while maintaining image quality, which is especially relevant because of increased MRI demands. Future research should address the effect of DLR on clinically relevant downstream tasks, including AI algorithms’ performances and biopsy decisions, and explore task-specific accelerated protocols for screening, image-guided biopsy, and treatment.

## 1. Introduction

The demand for magnetic resonance imaging (MRI) is increasing as its role in prostate cancer (PCa) early diagnosis, staging, active surveillance, follow-up, radiation treatment planning, and guidance expands [1]. This rise in demand is reflected in growing numbers of examinations, while overall waiting times for imaging services are increasing [2]. Including MRI in screening pathways would dramatically increase the demand for MRI capacity [3,4,5]. In the Goteborg-2 trial, second-stage MRI testing following PSA pre-selection included 6.7% and 17.9% of all invited men in the first round when PSA thresholds of 3.0 and 1.8 ng/mL were applied [3,6].

Multiple strategies can reduce the demand for MRI in diagnosis and screening, including using biomarkers [7]. Efficiency can be increased by acquiring only the necessary MRI sequences for the diagnostic task [8], such as omitting dynamic contrast-enhanced imaging [9,10]. Additional measures include accelerated MRI acquisition techniques, where time savings can lead to higher patient throughput [11]. Regardless of the strategies used, it is important to maintain or improve image quality and reduce the need for repeated acquisitions and patient recalls.

There are two main conventional accelerated MRI techniques: Parallel Imaging (PI) and Compressed Sensing (CS). PI uses the sensitivity profile of coil arrays to speed up image acquisition, while CS utilizes incoherent sampling of sparse information in MR images. Both techniques are commonly used in clinical applications, particularly T_2_w imaging [12]. Nevertheless, the signal-to-noise ratio and appearance of artifacts restrict the extent of acceleration achievable with these methods.

**Figure 1:**
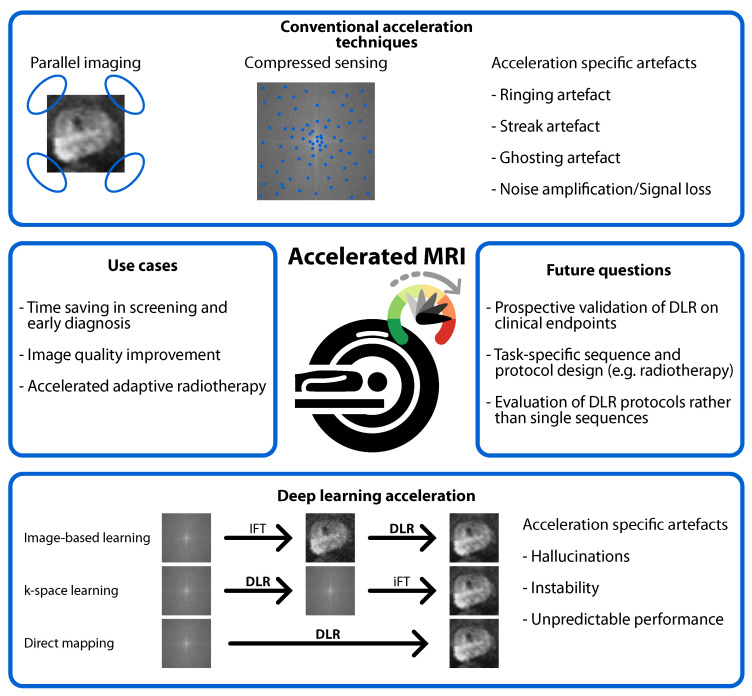
Visual summary of accelerated MRI in prostate imaging using conventional and deep learning reconstruction (DLR). Furthermore, potential use cases, challenges, and future questions for the implementation of accelerated prostate imaging protocols.

Deep learning reconstruction (DLR) is a technique that accelerates the process of MRI scanning by saving time, improving quality, and correcting motion [13]. DLR is offered by all major vendors of MR scanners [14] and can be used alongside conventional accelerated MRI methods. A notable aspect of DLR is its computational efficiency [15]. There are different approaches to implementing DLR in the reconstruction process. Image-based learning can improve the quality of reconstructed images after using conventional Inverse Fourier Transform (IFT) (Figure 1). K-space learning can enhance partially sampled raw data before reconstructions with IFT. Direct mapping achieves improvement and reconstruction in one step, using raw data as input to produce an improved image without IFT.

This review examines deep learning (DL)-based reconstruction approaches aimed at either accelerating the acquisition or enhancing the quality of prostate MRI. First, sequence-specific acceleration techniques are evaluated. Second, accelerated screening protocols are reviewed. Third, image quality metrics are analyzed. Finally, we discuss the opportunities and challenges associated with future trials utilizing DL acceleration for protocol personalization in MRI.

## 2. Materials and Methods

This systematic review was registered in the PROSPERO International Prospective Registry of Systematic Reviews (CRD420251032136), where the protocol is available, and follows the PRISMA-DTA (Preferred Reporting Items for Systematic Reviews and Meta-analysis for Diagnostic Test Accuracy) guidelines (Appendix A) [16,17]. The electronic databases PubMed, Web of Science, and Google Scholar were searched for primary publications in English assessing DLR for MRI of the prostate published up to 17 March 2025. The first 400 entries from Google Scholar were included. The search terms were: prostate AND MRI AND (deep learning OR DL OR artificial intelligence OR AI) AND (reconstruct* OR sequence).

Two researchers (SU and GA) screened titles, abstracts, and full texts for eligibility. References of included reports were searched for dataset completion. Discrepancies were resolved by consensus. Original reports using artificial intelligence (AI) to improve prostate MRI image quality and/or accelerate acquisition in humans were eligible for inclusion. Conference abstracts, case reports, and non-English reports were excluded. Contact with the authors was sought if reports were not available.

Information from the individual reports was transferred into a data collection form by one reviewer and verified by the second reviewer (Appendix B). Data were included as presented in the included studies. First, sequence-specific acceleration techniques were assessed. Second, accelerated screening protocols were reviewed. Third, image quality metrics were analyzed. Narrative synthesis was used to summarize the effects of DLR on image quality. The reduction in acquisition time achieved with DLR was summarized with descriptive statistics. Narrative synthesis summarized the diagnostic performance of human readers and AI with DLR. One reviewer performed and one verified risk of bias assessment using QUADAS-2.

Quality metrics comparing reference and test sequences were recorded in the data extraction instrument. Due to the high methodological heterogeneity of the studies and the use of numerous quality metrics (Appendix B), quantitative synthesis was restricted to comparing the subjective overall image quality among the studies. The subjective image quality ratings for every reconstruction were recorded (e.g., median and inter-quartile range, mean and standard deviation, or frequency tables). Additionally, the relative acquisition time of the DLR sequence relative to the conventional comparator was recorded.

Consistent with most of the included studies, image quality was treated as a pseudo-continuous variable. The within-group standardized mean difference served as the effect size, considering the varying number of levels in the Likert scales for quality assessment and the paired nature of observations. The correlation between the paired observations, necessary for estimating the standard error, was determined empirically as r = 0.85 on in-house data.

The effect size could not be calculated directly for studies reporting median and interquartile range. To increase the number of studies in a secondary meta-analysis, the means and standard deviations of image quality were computed for these studies as described by Wan et al. This method assumes a normal distribution of the measurements [18]. The inclusion of these additional studies was assessed using a sensitivity analysis. An additional sensitivity analysis was conducted, excluding studies at a high risk of bias.

A three-level, random-effects meta-analysis computed the summary effect size, taking multiple, non-independent observations on a single cohort into account. The meta-analysis was performed separately for T_2_w imaging and diffusion-weighted imaging (DWI) and implemented with the “metafor” package (Version 4.8-0) in R language for statistical computing (Version 4.4.1) [19]. A funnel plot visualized the risk of publication bias. The I^2^ statistic estimated the heterogeneity among the studies that was not attributable to random sampling. Appendix E includes a more detailed description of the statistical methods.

## 3. Results

**Figure 2:**
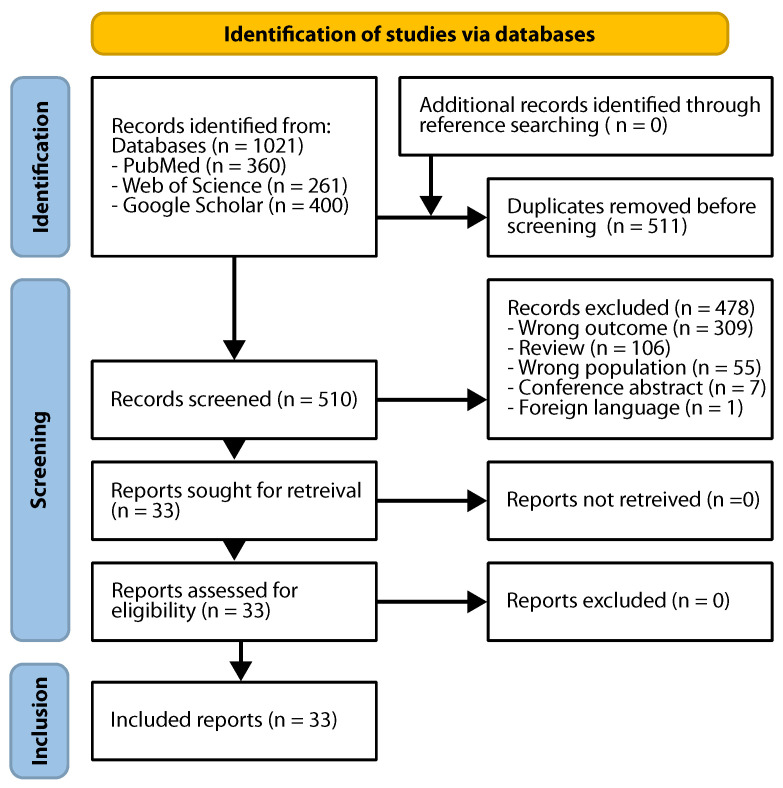
PRISMA study selection flowchart.

**Figure 3:**
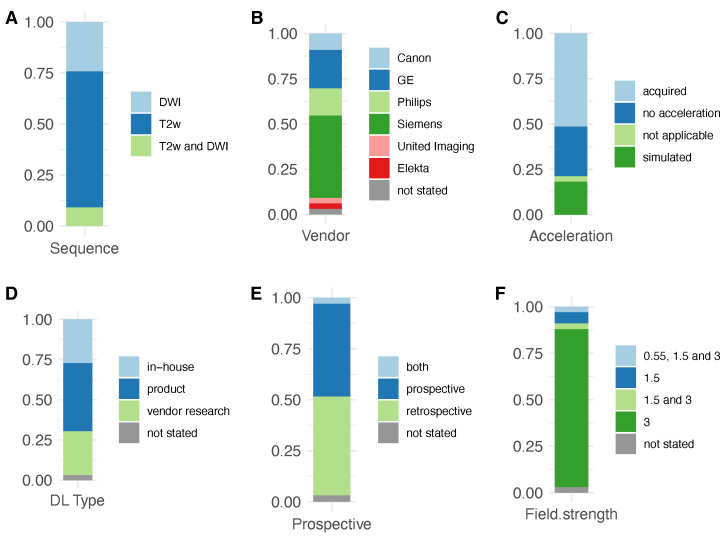
Characteristics of the included studies with (**A**) investigated sequence(s), (**B**) vendor of MRI scanners, (**C**) type of acceleration, (**D**) type of deep learning, (**E**) prospective or retrospective study design, and (**F**) MRI field strength.

The search identified 510 unique records, 478 of which were excluded. In particular, 309 investigated unsuitable outcomes, i.e., they did not assess the impact of deep learning image reconstruction on image quality or acquisition time. Examples include studies focusing on using AI for prostate cancer detection with MRI, image segmentation, radiomics analyses, or genomic profiling. Fifty-five studies were classified as evaluating the wrong population because they investigated conditions other than prostate cancer. Examples include imaging studies related to orthopedic, cerebral, or non-prostate pelvic diseases. Eventually, 33 studies (Figure 2) fulfilled the inclusion criteria, investigating possible image quality improvements for individual sequences [20,21,22,23,24,25,26,27,28,29,30,31,32,33,34,35,36,37,38,39,40,41,42,43,44,45,46,47,48,49,50,51,52], the application of DLR in abbreviated protocols [30,37,40], and the utility of DLR for radiotherapy [52]. Twenty-five reports investigated DLR for T_2_w imaging, while 11 assessed DLR for DWI (Figure 3). Studies reported on men undergoing MRI for PCa diagnosis (18), patients with biopsy-proven PCa (3), patients undergoing MRI for multiple indications (11), or healthy volunteers (1). One study [42] included publicly available data from the fastMRI initiative, a dataset of T_2_w and DWI containing the k-space data of 312 participants [53,54], complemented by data obtained from the vendor. The remaining 32 studies included only in-house data, with nine stating that the data were available from the authors upon request and one indicating that the data were unavailable.

Studies on T_2_w imaging and DWI investigated image-based learning (12), k-space learning (6), or direct mapping (14). Algorithms were developed in-house (9) and by vendors (23), 61% of which were product sequences. Studies most commonly employed 3T machines (85%) from a variety of vendors: Siemens (15), GE (7), Philips (5), Canon (3), United Imaging (1), and Elekta (1) (Figure 3).

### 3.1. Risk of Bias Assessment

Overall, the risk of bias was low, although seven studies were identified as having a high risk of bias or reduced applicability related to participant selection. This was due to the inclusion of participants with lesions visible on conventional MRI [28,39], those who had undergone biopsy or subsequently underwent prostatectomy [35,36,41,48], or healthy volunteers [44]. These cohorts were either enriched in patients with csPCa or consisted of younger participants with less csPCa. Therefore, they only partially represent clinical target populations. One study was at a high risk of bias in performing the reference test (conventional sequences) and index test (DLR sequences) [34]. The unblinded side-by-side comparison of the two sequences may result in expectation bias, where the readers favor one of the techniques, and anchoring bias, where previous observations influence subsequent evaluations. Appendix D includes the full risk of bias assessment.

### 3.2. Deep Learning Reconstruction

#### 3.2.1. DLR in T2w Imaging

DLR was employed to accelerate T_2_w imaging or improve the image quality sequences without additional acceleration. Nine studies applied DLR without additional acceleration compared to the reference T_2_w sequence. DLR achieved a higher image quality in seven reports, while two observed a lower quality. Seven studies used objective image quality metrics. The most commonly calculated image quality metrics were the SNR and the contrast-to-noise ratios (CNR) in three and four studies, respectively. One study used other metrics. The objective image quality improved in five studies, was unaltered in one study, and deteriorated in another study when acquisition times were constant.

DLR can potentially accelerate image acquisition while maintaining or increasing image quality. This was investigated with accelerated acquisitions (15) or simulations (3; Table 1). The acquisition time was reduced to a minimum of 13–64% of the reference sequence, representing a 1.56–7.7-fold acceleration. Acceleration did not lead to a deterioration in objective image quality metrics in any of the studies. Subjective preference at low acceleration factors favored DLR. At 3-fold and higher acceleration, DLR was of comparable quality to conventional sequences.

Six studies evaluated the effect of DLR on clinically relevant tasks. These included cancer detection by AI algorithms and human diagnostic performance, and detecting extraprostatic extension (EPE). AI algorithms trained on conventional MRI data performed worse on DL-reconstructed T_2_w data [47,50]. DLR had a less significant impact on human diagnosis, showing comparable performance for transition zone lesions, despite a 3-fold acceleration [35]. Another study with 10 patients showed similar diagnostic performance [30]. Evidence on EPE detection varied: one report showed similar performance despite a 37–70% reduction in acquisition time [35,41], while another showed decreased performance with high-resolution DLR T_2_w imaging [36].

Evaluation cohorts included a median of 46 participants (IQR 30–96). The study by van Lohuizen et al., a multicenter trial of three centers in the Netherlands, employed the largest testing cohort, with 306 participants [50]. It used reconstructed images and Fast Fourier Transform (FFT) to obtain sub-sampled k-space-like data that were sub-sampled, avoiding the prospective collection of k-space data. Some ADC values derived from DLR images varied from the reference but showed a strong linear correlation. Yet, these differences were typically modest and within the test–retest limits defined by the Quantitative Imaging Biomarkers Alliance (QIBA) profile 2024 [55].

#### 3.2.2. DLR in Diffusion-Weighted Imaging

Without additional acceleration, the subjective image quality of DLR DWI was comparable in three studies and improved in two studies. All five studies found a higher signal-to-noise ratio (SNR) in DLR images (1.24–2.17-fold). The studies had no other shared objective image quality metric.

DLR was employed to accelerate image acquisition in eight studies. Acquisition time reductions ranged between 13% and 69% of the reference sequences. The subjective image quality was independent of the degree of acceleration. However, retrospective sub-sampling of k-space simulated acceleration in five studies, and only three studies acquired accelerated sequences, potentially reducing gains in image quality from reduced motion artifacts. The effects of DLR in DWI on downstream tasks remain unexplored. Models were evaluated on relatively small cohorts with a median of 51 participants (IQR 36–60), lowering clinical generalizability.

#### 3.2.3. DLR in AI-Accelerated Screening/Diagnostic Protocols

Three studies investigated complete accelerated bi-parametric protocols [30,37,40], comparable to proposed screening protocols.

In a retrospective study, Johnson et al. simulated undersampling of axial and coronal T_2_w and axial DWI sequences, validating a simulated acceleration from 11:48 min to 3:12 min (− 73%) at maintained image quality in 20 participants. No difference in diagnostic performance was detected, albeit only a few lesions were present in the cohort [30].

Lee et al. conducted a retrospective analysis of 40 prospectively recruited patients who underwent axial T_2_w and DWI. Standard of care imaging, T_2_w with a reduced number of averages and standard or higher resolution, and DWI with a reduced number of averages were reconstructed with different DLR strengths. With all of the sequences, DLR increased SNR and CNR ratios. Two radiologists rated the overall image quality as similar to the standard of care for most reconstructions. Only the accelerated T_2_w sequence reconstructed with medium-strength DLR showed a higher quality [37].

Oerther et al. studied 77 men suspected of prostate cancer using an accelerated and abbreviated bi-parametric MRI protocol with T_2_w imaging and DWI (3:28 min) compared to standard mpMRI with 3-planar T_2_w TSE and DCE (25:45 min). The abbreviated protocol had better image quality, higher inter-rater agreement in PI-RADS 2.1 scoring, and comparable diagnostic performance to the standard protocol [40].

#### 3.2.4. DLR in PI-RADS Assessment

Thirteen studies assessed the effect of DLR on PI-RADS scoring, including studies investigating bi-parametric screening protocols (DLR T_2_w and DLR DWI; 3), DLR T_2_w with conventional DWI (9), or DLR DWI with conventional T_2_w (1). Most studies found good-to-excellent agreement or no significant difference between PI-RADS scoring performed on conventional and DLR images. Only one study observed significant upgrading with DLR DWI [29]. Inter-reader agreement increased with DLR in three studies [25,29,50]. In a prospective study of DLR for T_2_w imaging, Bischoff et al. observed a change in PI-RADS scores in 6% of patients that reduced the number of PI-RADS 3 cases by 78–86% through up- or down-scoring [21].

In a study comparing conventional multi-parametric MRI to DLR bi-parametric MRI, one of two readers had a lower specificity for lesions ≥ PI-RADS 3 and a lower sensitivity for lesions ≥ PI-RADS 4 on DLR bi-parametric MRI [43]. Only one study offered sufficient details to calculate an effect size [21]. Therefore, a meta-analysis of diagnostic performance was not possible.

### 3.3. Methods for Image Quality Assessment

#### 3.3.1. Objective Image Quality Metrics

Twenty-six studies employed objective image quality metrics. However, the specific metrics used in the studies varied widely (Table 2). Image quality metrics most commonly included SNR, CNR, and edge rise distance/slope profile in 13, 12, and 6 studies, respectively. The most commonly used fully referenced quality metrics were structural similarity index, peak SNR, and root mean square error. Those metrics compare DLR images against a high-quality ground truth.

#### 3.3.2. Subjective Image Quality Metrics

Twenty-nine studies assessed subjective image quality with one to five readers (median 2). Quality metrics, while differently defined, fell into one of six categories: overall image quality, noise, artifacts, sharpness, conspicuity of organs, and diagnostic confidence. Binary classifications, 4- and 5-tier Likert classifications were used.

Appendix B lists all subjective and objective image quality metrics for individual studies.

### 3.4. Quantitative Synthesis

Twenty-six studies reported subjective overall image quality and were potentially eligible for inclusion in the meta-analysis. Five studies did not report sufficient details to obtain an effect size. Two studies were excluded because they reported an interquartile range of 0, which did not allow for approximating a standard error. The final meta-analysis included eight studies in the primary analysis. An additional 11 studies with effect sizes computed indirectly were included in the extended cohort for the sensitivity analysis (Appendix F). The studies reported 26 comparisons in the primary and 52 in the extended analysis. One study reported a combined quality score for T_2_w imaging and DWI included in the meta-analysis of both sequences.

**Figure 4:**
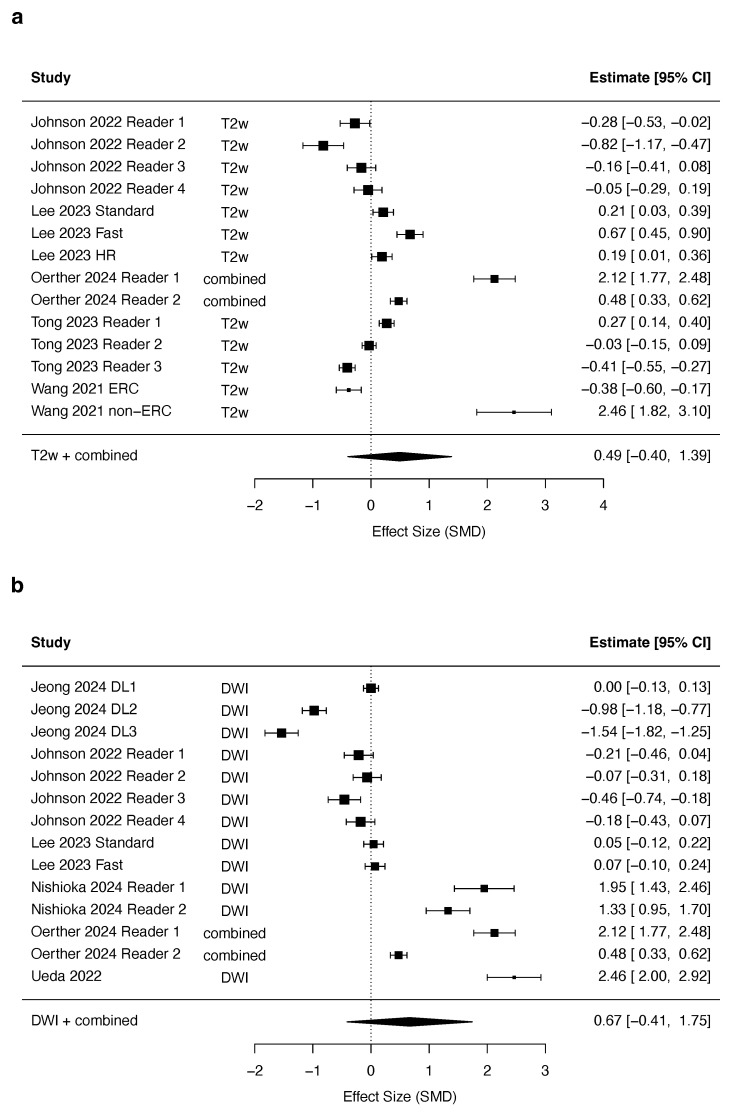
Forest plot of studies investigating DLR for T_2_w imaging (**a**) and DWI (**b**) [29,30,37,39,40,47,48,51].

**Figure 5:**
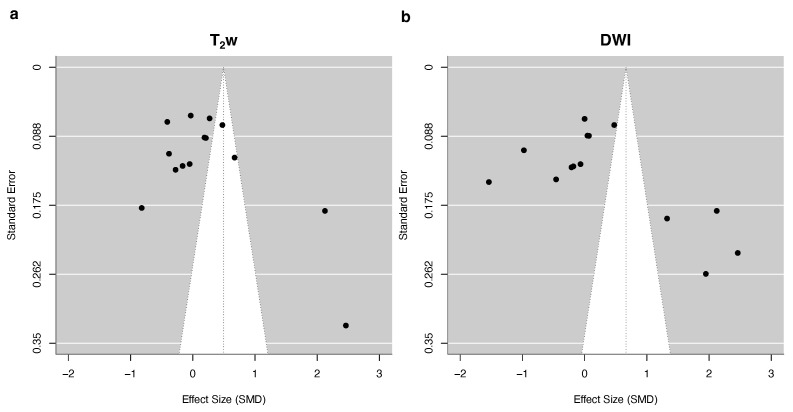
Funnel plots of the studies included in the meta-analysis of subjective image quality in T_2_w (**a**) and DWI (**b**).

**Figure 6:**
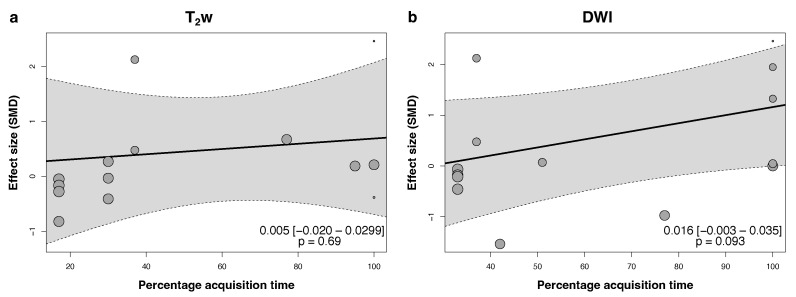
Bubble plot of the meta-regression examining the association between subjective image quality and the relative acquisition time for T_2_w (**a**) and DWI (**b**). Each bubble represents a study, and its size is proportional to the precision of the effect size estimate. The 95% confidence interval of the regression line is shaded in gray.

The meta-analysis revealed a non-significant trend towards a higher image quality with DLR on T_2_w imaging (summary effect size of 0.49, 95% confidence interval −0.39–1.39, *p* = 0.25, df = 13, Figure 4a). However, the overall heterogeneity of between-study comparisons was high, with an I^2^ of 77%. The heterogeneity of within-study comparisons was moderate at I^2^ = 22%, resulting in a total heterogeneity of I^2^ = 98%. The funnel plot reveals some asymmetry favoring DLR, indicative of possible publication bias (Figure 5). The sensitivity analysis, including studies where the effect size was approximated from median and interquartile range, showed a borderline significant trend towards higher image quality for DLR (summary effect size of 1.38, 95% confidence interval 0.00–2.04, *p* = 0.05, df = 32). However, this increase in quality was determined by two studies with extreme results (Appendix F). Image quality was not associated with the relative acquisition time (Figure 6a).

The analysis of DWI revealed no significant association of image quality with the reconstruction method (summary effect size of 0.67, 95% confidence interval −0.41–1.74, *p* = 0.21, df = 13, Figure 4b). The overall heterogeneity of between-study comparisons was high, with an I^2^ of 78%. The heterogeneity of within-study comparisons was moderate at I^2^ = 20%, resulting in a total heterogeneity of I^2^ = 99%. The funnel plot showed a moderate asymmetry favoring DLR (Figure 5), indicative of possible publication bias. The sensitivity analysis, including studies where the effect size was approximated from median and interquartile range, did not change the results (summary effect size of 0.62, 95% confidence interval −0.26–1.50, *p* = 0.16, df = 19, Appendix F). Meta-regression showed no significant association between the relative acquisition time and image quality Figure 6.

## 4. Discussion

This systematic review evaluated the rapidly increasing literature on DLR for prostate MRI. Most of the research took place in the screening and early diagnostic setting. However, cohorts were frequently heterogeneous. The assessment of DLR primarily focused on image quality evaluation, and research on the effects of DLR on downstream tasks is only just beginning to accrue. Methodological heterogeneity and considerable variability in quality metrics limited the scope of quantitative comparisons. The potential of DLR for prostate imaging has been recognized and reaches beyond diagnostic imaging with initial research on the use of DLR for treatment guidance in radiotherapy.

### 4.1. Benefits of DLR

DLR can impact MRI protocol development and aid in creating MRI screening strategies for prostate cancer. This review demonstrates that DLR can speed up T_2_w and DWI acquisition of the prostate by up to 8-fold without sacrificing image quality [50]. A meta-analysis of the subjective image quality revealed no significant difference between conventional and DLR images among the included studies. However, there was considerable heterogeneity among the studies, with some aiming for image quality improvement and some for accelerated scanning time. Faster scans improve patient comfort and reduce motion artifacts. DLR can enhance image quality on lower-specification MRI systems without longer acquisition times. Cost-effectiveness studies indicate that DLR increases scanner capacity more effectively than buying another MRI system or extending weekend hours [11].

### 4.2. Risks of DLR

**Figure 7:**
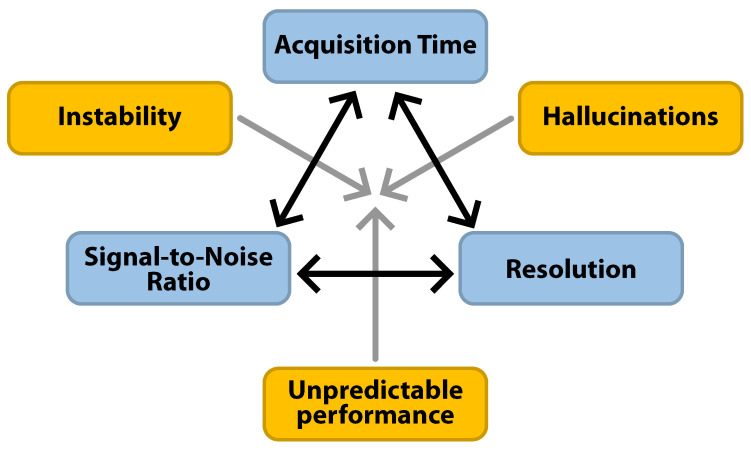
Classically, image quality was seen as a trade-off between the signal-to-noise ratio, acquisition time, and spatial resolution (blue). Deep learning reconstruction (DLR) may increase image quality while reducing the acquisition time. However, as the sampled data become increasingly sparse, hallucinations, over-smoothing, and instability (yellow) become a greater concern.

While image quality in conventional reconstruction was a trade-off between acquisition time, SNR, and resolution, additional factors play into DLR (Figure 7). One key risk is hallucinations, the addition or removal of realistic-looking image details, which may arise when there are multiple plausible solutions to the inverse image reconstruction problem. A deep learning model, influenced by the priors learned from the training data, may favor solutions introducing hallucinated features. The risk of hallucinations increases with stronger acceleration, i.e., decreasing sampling rates [56]. Reduced image noise may also reveal artifacts that are not ordinarily visible, leading to false-positive findings. A second phenomenon is instability, where small perturbations in the input (e.g., noise) lead to severe artifacts or degradation of image quality [57]. Importantly, hallucination and instability are inevitable in many cases of DLR. Achieving perfect accuracy, stability, and hallucination-free reconstructions simultaneously is not possible [58]. Nonetheless, DLR and conventional acceleration techniques require similar caution in their clinical implementation.

Further artifacts specific to DLR include over-smoothing, which can mimic the charcoal sign in the transition zone, leading to false positives. Conversely, the loss of subtle findings adjacent to the capsule may cause false negatives. These artifacts depend on algorithm settings, vendors, scanners, and patient anatomy.

### 4.3. Effect of DLR on PI-RADS Assessment and Downstream Clinical Tasks

Image quality is only a surrogate marker for the aim of imaging: improving outcomes in a diagnostic or therapeutic pathway. Therefore, it is essential to evaluate the effect of DLR on downstream tasks such as the diagnosis of prostate cancer, AI-assisted lesion detection, biopsy guidance, or automated segmentation for radiotherapy (Figure 1). Initiatives to probe the effect of DLR on downstream tasks were ongoing.

There was no evidence that DLR would significantly impact PI-RADS scoring for human readers. The agreement between conventional and DLR images is generally high, and the significant difference in one study was modest in magnitude [40]. As only one comparison used DLR in T_2_w imaging and DWI simultaneously, data on fully DLR protocols are still scarce. Nonetheless, no evidence suggested that DLR significantly impacts PI-RADS scoring.

It is also relevant to go beyond comparing two imaging outcomes (e.g., PI-RADS). Instead, we should relate outcomes from imaging to a reference standard, e.g., follow-up or histological diagnosis of PCa. Among the studies that evaluated DLR on a downstream task not based on an imaging outcome (e.g., detection of histologically proven cancer or extraprostatic extension), the performance of conventional and DLR images was similar [29,30,34,35,41,48]. Where significant differences occurred, they were generally small [36]. This non-inferior evidence must still be considered preliminary, as the studies were retrospective, often included small patient cohorts, were at a high or uncertain risk of selection bias, and used biopsies guided by standard MRI as the reference standard. Biopsy guidance with DLR sequences remains unexplored.

ADC values derived from DLR may exhibit slight deviations from those obtained using conventional imaging techniques. These differences can be attributed to several factors: (1) denoising effects, where noise reduction—particularly at high b-values—can unmask underlying signal; (2) the characteristics of the DLR algorithm and its training data; (3) variations in sequence design and imaging protocols; and (4) tissue-specific effects. While the clinical significance of these differences is likely limited, given that relative ADC contrasts between tissues are generally preserved and the absolute changes are minor, sequence-specific validation is advisable before quantitative analysis.

### 4.4. Effect of DLR on Diagnostic AI

Diagnostic AI for PCa detection on MRI has been a focus of intense research, and models for clinical use are available from multiple vendors. Therefore, the combined diagnostic performance of DLR images as input for diagnostic AI algorithms is of great importance.

Van Lohuizen et al. and Tong et al. investigated the effect of DLR of T_2_w images on diagnostic prostate AI [47,50]. While on objective and subjective metrics, image quality was higher than or comparable to the reference standard in both studies, the performance of diagnostic AI on DLR images was inferior. This highlights the challenge posed by the out-of-domain nature of DLR images. One solution is to incorporate DLR images into the training data for AI. Nonetheless, use cases of AI where multiple AI algorithms interact may lead to unexpected outcomes (the double black box phenomenon), warranting careful evaluation in clinical practice.

### 4.5. Heterogeneity in the Evaluation of DLR Image Quality

Various metrics were employed for subjective and objective image quality assessment, limiting the comparability of the results across the studies included in this systematic review. An objective quantification of image quality is attractive as it may allow a comparison of outcomes across different DLR algorithms. However, the apparent precision of a numerical metric may correlate only poorly with the visual quality of an image. The SNR and CNR were the most common quality metrics. While both relate to image noise and signal, they neglect sharpness and image distortions. Similarly, the most common fully referenced metrics, peak SNR and structural similarity index measure (SSIM), agree poorly with radiologists’ subjective image quality assessments [59]. Furthermore, they are strongly dependent on image normalization [60].

No single image quality metric is sufficiently sensitive to all possible causes of image distortions. In response to this, Dohmen et al. suggested a combination of SSIM, learned perceptual image patch similarity (LPIPS), mean squared error (MSE), and normalized mutual information (NMI) to cover a large range of possible distortions [60]. Still, using fully referenced metrics will be unable to detect improvements in the quality of DLR images compared to the reference standard.

### 4.6. DLR Beyond Diagnosis

The increasing demand for prostate MRIs will likely require shorter and more personalized protocols that will have to be adapted more closely to downstream tasks. Consequently, novel sequences need to be evaluated in concert with the intended use case. For example, a diagnostic protocol requires high sensitivity to identify clinically significant cancer. Meanwhile, a simulation/planning scan for radiation oncology might weigh accurate anatomical representation higher, as this will impact treatment planning and dose delivery to known lesions. Finally, MRI for adaptive radiotherapy serves as the confirmation of existing treatment plans. Therefore, radiation oncologists may accept a slightly reduced image quality if the shorter acquisition time reduces patient motion and discomfort.

### 4.7. Limitations

This systematic review has some limitations. The heterogeneity among the included studies restricted the scope of the quantitative synthesis to the subjective overall image quality. This analysis confirmed the high methodological heterogeneity among the studies. Studies varied in terms of the included patient population, the operating point of the AI models (quality improvement vs. acceleration), and metrics for image quality assessment. Additional variation was introduced by different scanners from different vendors, with variable field strengths, and different clinical outcomes. Such variation represents the heterogeneity among medical centers, as the centers’ role in the healthcare system, long-term infrastructure investment, and software/hardware integration differ. It is also representative of a multi-faceted challenge where image quality is only the first layer. Only a few studies investigated the impact of DLR on the diagnostic performance of prostate MRI or biopsy guidance as clinically relevant endpoints. Finally, the review only included studies published in English. This decision was based on the limited proficiency of the research team in other relevant languages, which would have hindered devising a search strategy and assessing manuscripts consistently and reliably across languages.

### 4.8. Summary and Recommendations

Box 1 summarizes the principal findings of this systematic review. Box 2 presents recommendations and suggestions for the implementation of DLR for prostate MRI based on the findings of this systematic review.

Box 1 **Principal findings of the systematic review.**

**Image quality in T_2_w and DWI sequences**

- Non-accelerated DLR improves the subjective and objective image quality of T_2_w sequences, with maintained quality at 3-fold and stronger acceleration.
- DLR achieves a higher SNR in DWI sequences.
- Diagnostic AI performs invariably less well on DLR images, even if objective and subjective image quality metrics were higher.

**DLR screening protocols and diagnostic performance**

- DLR may reduce acquisition times without a loss in image quality.
- PI-RADS scoring is comparable between conventional and DLR sequences.
- Bi-parametric DLR protocols show similar diagnostic accuracy in small study cohorts.

**Methods image quality assessment**

- Common image quality metrics include no-reference and fully-referenced metrics (structural similarity index, peak SNR, and root mean square error).
- Subjective image quality metrics assess overall image quality, noise, artifacts, sharpness, conspicuity of structures, and diagnostic confidence.

**Risks and limitations of DLR**

- Stronger acceleration increases the risk of hallucinations and instability.
- Perfect accuracy, stability, and hallucination-free reconstructions are not achievable with DLR, requiring caution in clinical implementation.

Box 2 **Recommendations and suggestions on the implementation of DLR.**

**Recommendations:**

- Select the acceleration factor of DLR judiciously to balance the desired acquisition time savings with the risk of hallucinations and instability [56].
- Implement deep learning image reconstruction in coordination with the entire clinical team, including referring physicians if necessary, e.g., by means of side-by-side review sessions.
- Monitor the compatibility of DLR with downstream workflows such as diagnostic AI [47,50].
- Adapt DLR to the specific clinical context. The trade-off between image quality and acquisition time may vary between use cases [52].
- Monitor diagnostic performance and biopsy yield, especially when biopsy decision making transitions from conventional to DLR sequences [21].

**Considerations:**

- Implement DLR in a step-wise fashion rather than full DLR protocols.
- Implement DLR in addition to conventional sequences initially and while fine-tuning sequence parameters to achieve the desired diagnostic quality.
- Fully replace secondary sequences (coronal and sagittal T_2_w, axial T_1_w and DCE) before replacing axial T_2_w and DWI.
- Standardize parameters for image quality assessment or find a consensus on which one should be used.
- Investigate the effect DLR would have had on biopsy decisions in patients undergoing conventional and DLR imaging.
- Perform a comparative analysis of diagnostic AI using conventional and DLR-acquired images to assess consistency and potential performance shifts.
- Record the use of DLR in reports to facilitate audits and AI monitoring.

## 5. Conclusions

Image quality analyses indicate that DLR can accelerate high-quality prostate MRI in various use cases. Future research should prioritize prospective, reader/blinded, and paired study designs with histological validation as a reference standard to determine the effect of DLR on diagnostic accuracy. Developing task-specific protocols will be key to successfully implementing DLR, particularly when additional AI models are applied to images further downstream in the analysis pathway.

## Figures and Tables

**Table 1:** Summary of acceleration approach (simulated or acquired) and domain of the input data (image space, k-space, or k-space with direct mapping).

	T_2_w			DWI		
	**Image**	**k-Space**	**Direct Mapping**	**Image**	**k-Space**	**Direct Mapping**
Simulated	2	0	1	2	1	2
Acquired	3	2	10	0	0	2
Not applicable	4	3	0	2	1	1

**Table 2:** Summary of objective and subjective image quality metrics used in the included studies.

Objective Image Quality Metrics	Subjective Image Quality Metrics
- Signal-to-noise ratio (15)	- Overall image quality
- Contrast-to-noise ratio (13)	- Noise
- Edge rise distance / Slope profile (6)	- Artifacts
- Structural similarity index (6)	- Sharpness
- Peak signal-to-noise ratio (5)	- Conspicuity
- Root mean squared error (4)	- Diagnostic confidence
- Performance of diagnostic AI (3)	
- Feature similarity index (1)	
- Perceptual index (1)	
- Variance (1)	
- Gaussian log likelihood (1)	
- Edge keeping index (1)	

**Figure A1:**
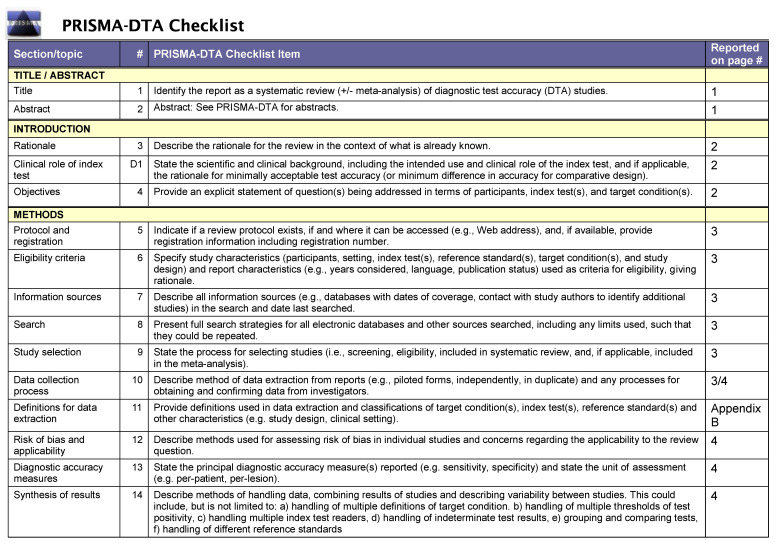

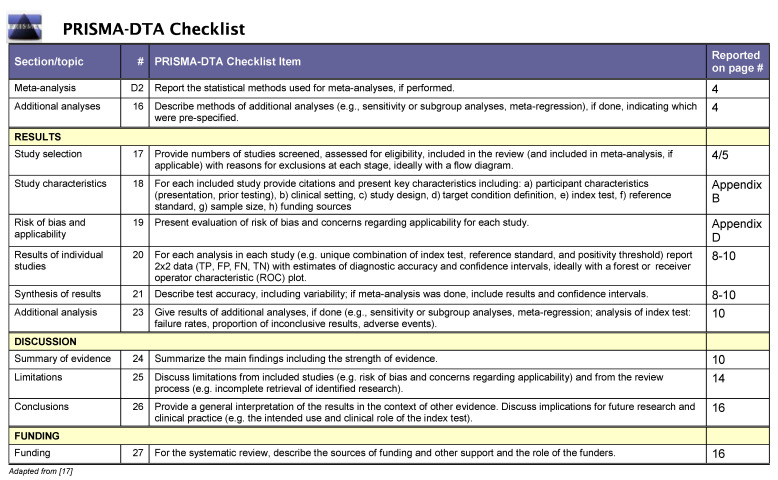
PRISMA Checklist.

**Figure A2:**
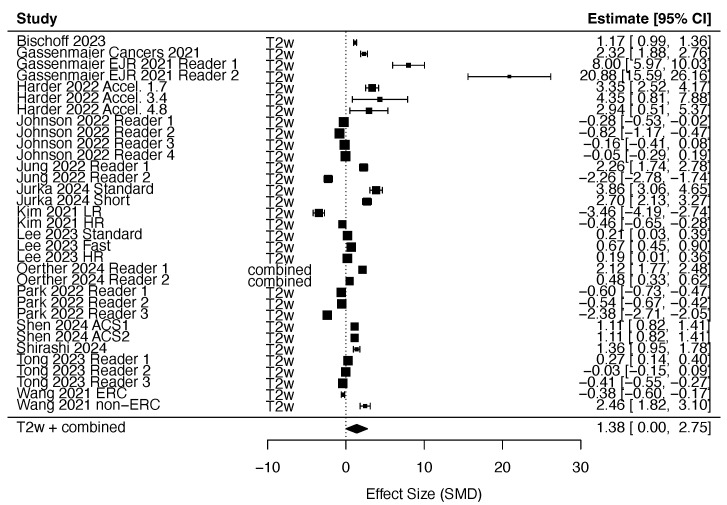
Forest plot of all studies investigating subjective image quality of DLR for T_2_w sequences, including studies where the effect size had to be approximated from the median and interquartile range.

**Figure A3:**
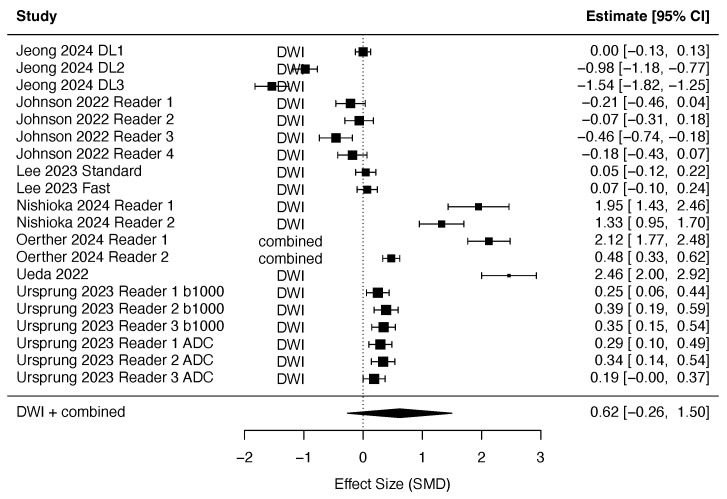
Forest plot of all studies investigating subjective image quality of DLR for DWI sequences, including studies where the effect size had to be approximated from the median and interquartile range.

## Data Availability

No new data were created or analyzed in this study. Data sharing is not applicable to this article.

## Abbreviations

The following abbreviations are used in this manuscript:

ADC	Apparent diffusion coefficient
AI	Artificial intelligence
CNR	Contrast-to-noise ratio
CS	Compressed sensing
DLR	Deep learning reconstruction
DWI	Diffusion-weighted imaging
EPE	Extra-prostatic extension
IFT	Inverse Fourier transform
IQR	Interquartile range
(mp)MRI	(multi-parametric) Magnetic resonance imaging
PCa	Prostate cancer
PI	Parallel imaging
PI-RADS	Prostate imaging reporting and data system
PSA	Prostate-specific antigen
SNR	Signal-to-noise ratio
T_2_w	T_2_-weighted

## Appendix B. Data Extraction Instrument

**Table A1:** Data extraction instrument.

#	First Author	Journal	Year	Vendor	Field Strength
1	Belue	European Journal of Radiology	2024	Philips	3
2	Bischoff	Radiology	2023	Philips	3
3	Boschheidgen	Magnetic Resonance Imaging	2025	Siemens	3
4	Cochran	Clinical Imaging	2025	GE	3
5	Gassenmaier	Cancers	2023	Siemens	3
6	Gassenmaier	European Journal of Radiology	2021	Siemens	3
7	Gassenmaier	Cancers	2021	Siemens	3
8	Harder	Cancers	2022	Philips	3
9	Hu	Frontiers in Oncology	2021	Siemens	3
10	Jeong	Quantitative imagin in medicine and surgery	2024	Siemens	3
11	Johnson	Journal of Magnetic Resonance Imaging	2022	Siemens	3
12	Jung	British Journal of Radiology	2022	Siemens	3
13	Jurka	Quantitative imagin in medicine and surgery	2024	Philips	3
14	Kaye	Radiology: Artificial Intelligence	2020	GE	3
15	Kim	Cancers	2024	GE	3
16	Kim	Nature Scientifc Reports	2024	Siemens	3
17	Kim	European Journal of Radiology	2021	Siemens	3
18	Lee	European Journal of Radiology	2023	GE	3
19	Liu	Journal of Digital Imaging	2021	Siemens	3
20	Nishioka	European Journal of Radiology Open	2024	Philips	3
21	Oerther	European Radiology	2024	Siemens	3
22	Park	Journal of Magnetic Resonance Imaging	2022	GE	3
23	Pfaff	Nature Scientifc Reports	2024	Siemens	0.55, 1.5 and 3
24	Riederer	Abdominal Radiology	2024	GE	3
25	Sato	Radiological Physics and Technology	2024	Canon	1.5
26	Shen	Academic Radiology	2024	United Imaging	3
27	Shirashi	Magnetic Resonance Imaging	2024	Canon	3
28	Tong	Journal of Magnetic Resonance Imaging	2023	Siemens	3
29	Ueda	Radiology	2022	Canon	3
30	Ursprung	European Journal of Radiology	2023	Siemens	3
31	van Lohuizen	European Radiology	2024	NA	NA
32	Wang	Abdominal Radiology	2021	GE	1.5 and 3
33	Zhu	Radiation Oncology	2023	Elekta	1.5

**Table A1:** Cont.

#	Sequence	DL Type	Space	Direct Mapping	Participants
1 Belue	T_2_w	in-house	Image	NA	96
2 Bischoff	T_2_w	product	k-space	yes	109
3 Boschheidgen	T_2_w	product	k-space	yes	120
4 Cochran	DWI	product	k-space	no	52
5 Gassenmaier	T_2_w	vendor research	k-space	yes	30
6 Gassenmaier	T_2_w	vendor research	k-space	yes	30
7 Gassenmaier	T_2_w	vendor research	k-space	yes	60
8 Harder	T_2_w	product	k-space	yes	33
9 Hu	DWI	in-house	Image	NA	Total 210, validation 60
10 Jeong	DWI	vendor research	k-space	yes	70
11 Johnson	T_2_w and DWI	in-house	k-space	yes	Total 113, validation 20
12 Jung	T_2_w	in-house	Image	NA	40
13 Jurka	T_2_w	vendor research	k-space	yes	47
14 Kaye	DWI	in-house	Image	NA	Total 140, validation 37
15 Kim	T_2_w	product	k-space	no	88
16 Kim	T_2_w	NA	NA	NA	162
17 Kim	T_2_w	vendor research	k-space	yes	46
18 Lee	T_2_w and DWI	product	k-space	no	40
19 Liu	T_2_w	in-house	Image	NA	346
20 Nishioka	DWI	product	k-space	yes	32
21 Oerther	T_2_w and DWI	product	k-space	yes	77
22 Park	T_2_w	product	k-space	no	109
23 Pfaff	DWI	in-house	Image	NA	409 training, 112 validation, 51 testing
24 Riederer	T_2_w	product	k-space	no	17
25 Sato	T_2_w	product	Image	NA	13
26 Shen	T_2_w	product	Image	NA	40
27 Shirashi	T_2_w	vendor research	Image	NA	28
28 Tong	T_2_w	vendor research	k-space	yes	80
29 Ueda	DWI	product	Image	NA	60
30 Ursprung	DWI	vendor research	k-space	yes	35
31 van Lohuizen	T_2_w	in-house	Image	NA	Total 1536, validation 306
32 Wang	T_2_w	product	k-space	no	31
33 Zhu	T_2_w	in-house	Image	NA	19

**Table A1:** Cont.

#	Acceleration	Study Design	Clinical Setting
1 Belue	NA	retrospective	Diagnostic prostate MRI
2 Bischoff	acquired	prospective	Diagnostic prostate MRI
3 Boschheidgen	acquired	prospective	Diagnostic prostate MRI
4 Cochran	simulated k-space	retrospective	Diagnostic prostate MRI
5 Gassenmaier	acquired	prospective	Diagnostic or biochemical recurrence
6 Gassenmaier	acquired	retrospective	Diagnostic prostate MRI
7 Gassenmaier	acquired	prospective	Diagnostic prostate MRI
8 Harder	acquired	prospective	Biopsy proven prostate cancer
9 Hu	NA	prospective	Diagnostic prostate MRI
10 Jeong	acquired	retrospective	Biopsy proven prostate cancer
11 Johnson	simulated k-space	retrospective	Any prostate MRI
12 Jung	acquired and simulated	both	Diagnostic prostate MRI
13 Jurka	acquired	prospective	Diagnostic prostate MRI
14 Kaye	simulated k-space	retrospective	Any prostate MRI
15 Kim	NA	prospective	Diagnostic prostate MRI
16 Kim	acquired	retrospective	Diagnostic prostate MRI
17 Kim	acquired	retrospective	Any prostate MRI
18 Lee	acquired	prospective	Any prostate MRI
19 Liu	NA	unclear	Diagnostic prostate MRI
20 Nishioka	NA	retrospective	Diagnostic prostate MRI
21 Oerther	acquired	prospective	Diagnostic prostate MRI
22 Park	acquired	retrospective	Diagnostic prostate MRI
23 Pfaff	simulated k-space	retrospective	Healthy volunteers + any prostate MRI
24 Riederer	NA	prospective	Any prostate MRI
25 Sato	NA	prospective	Healthy volunteers
26 Shen	acquired	prospective	Any prostate MRI
27 Shirashi	NA	retrospective	Any prostate MRI
28 Tong	acquired	retrospective	Diagnostic or AS prostate MRI
29 Ueda	NA	retrospective	Diagnostic prostate MRI
30 Ursprung	simulated k-space	retrospective	Any prostate MRI
31 van Lohuizen	simulated k-like space after FFT	retrospective	Diagnostic prostate MRI
32 Wang	NA	prospective	Diagnostic prostate MRI
33 Zhu	acquired	prospective	Radiotherapy MRI for adaptive treatment

**Table A1:** Cont.

#	# Readers	Acquisition Time Reduction
1 Belue	5	100%
2 Bischoff	2	64%
3 Boschheidgen	2	56–72%
4 Cochran	2	32–100%
5 Gassenmaier	2	110%
6 Gassenmaier	2	35%
7 Gassenmaier	2	37%
8 Harder	4	41–100%
9 Hu	1	not specified
10 Jeong	2	42–100%
11 Johnson	4	18–27%
12 Jung	2	29–58%
13 Jurka	3	50%
14 Kaye	2	13%
15 Kim	2	100%
16 Kim	2	31%
17 Kim	2	24–31%
18 Lee	2	For DWI: 51–100%, For T_2_w 67–100%
19 Liu	1	100%
20 Nishioka	2	100%
21 Oerther	2	37%
22 Park	3	63%
23 Pfaff	NA	58%
24 Riederer	3	100%
25 Sato	2	100%
26 Shen	2	47–53%
27 Shirashi	2	100%
28 Tong	3	30–50%
29 Ueda	2	100%
30 Ursprung	3	69%
31 van Lohuizen	NA	13–25%
32 Wang	3	100%
33 Zhu	NA	28%

**Table A1:** Cont.

#	Subjective Image Quality Metrics								
	Motion	Artifacts	Sharpness	Noise	Lesion Conspicuity	Structural Conspicuity	Diagnostic Confidence	Overall Quality	Others
1 Belue	X			X				X	
2 Bischoff		X	X		X	X		X	
3 Boschheidgen	X		X	X			X		
4 Cochran		X	X	X			X	X	
5 Gassenmaier		X	X	X	X		X	X	
6 Gassenmaier		X	X	X	X		X	X	
7 Gassenmaier		X		X	X		X	X	Natural impression
8 Harder	X		X	X	X		X	X	
9 Hu								X	
10 Jeong				X	X			X	
11 Johnson		X				X		X	
12 Jung		X	X					X	Perceived SNR
13 Jurka	X		X	X		X		X	Contrast
14 Kaye			X	X	X	X		X	
15 Kim		X		X		X			
16 Kim									
17 Kim		X	X		X			X	
18 Lee		X						X	
19 Liu						X			
20 Nishioka					X	X		X	
21 Oerther				X		X		X	
22 Park					X			X	
23 Pfaff									
24 Riederer		X	X		X	X		X	Subjective SNR
25 Sato		X		X		X		X	
26 Shen		X	X		X	X		X	
27 Shirashi		X	X	X				X	Conrast
28 Tong						X		X	
29 Ueda								X	
30 Ursprung		X	X	X			X	X	
31 van Lohuizen									
32 Wang		X			X			X	
33 Zhu									

Studies reporting a subjective image quality metrics are marked with ’X’.

**Table A1:** Cont.

#	Objective Image Quality Metrics							
	SNR	CNR	Intensity Gradient Metric	SSIM	pSNR	RMSE	AI	Others
1 Belue							X	
2 Bischoff	X	X	X					
3 Boschheidgen	X	X	X					
4 Cochran	X							
5 Gassenmaier								
6 Gassenmaier								
7 Gassenmaier								
8 Harder	X	X	X					
9 Hu				X	X	X		Feature similarity index
10 Jeong	X	X						
11 Johnson								
12 Jung				X	X	X		
13 Jurka	X		X					
14 Kaye				X		X		
15 Kim	X	X						
16 Kim								
17 Kim	X	X						
18 Lee	X	X						
19 Liu				X	X	X		Perceptual index
20 Nishioka	X	X						
21 Oerther								
22 Park	X	X						
23 Pfaff								Variance, Gaussian log-likelihood
24 Riederer								
25 Sato	X	X						
26 Shen	X	X	X					
27 Shirashi		X	X					Contrast
28 Tong							X	
29 Ueda	X	X						
30 Ursprung	X							
31 van Lohuizen				X	X		X	
32 Wang								Signal ratio
33 Zhu				X	X			MAE, edge keeping distance

Abbreviations: CNR: contrast-to-noise ratio, DWI: diffusion-weighted imaging, MAE: mean absolute error, (p)SNR: (peak) signal-to-noise ratio, RMSE: root mean square error, SSIM: structural similarity index, T_2_w: T2-weighted. Studies reporting an objective image quality metrics are marked with ’X’.

## Appendix C. Study Categorization

**Table A2:** Categorization of the included studies.

Data Space		
Image	k-Space	k-Space w/ Direct Mapping
1 Belue	4 Cochran	2 Bischoff
9 Hu	15 Kim	3 Boschheidgen
12 Jung	18 Lee	5 Gassenmaier
14 Kaye	22 Park	6 Gassenmaier
19 Liu	24 Riederer	7 Gassenmaier
23 Pfaff	32 Wang	8 Harder
25 Sato		10 Jeong
26 Shen		11 Johnson
27 Shirashi		13 Jurka
29 Ueda		17 Kim
31 van Lohuizen		20 Nishioka
33 Zhu		21 Oerther
		28 Tong
		30 Ursprung

**Table A2:** Cont.

Sequence		
T_2_w	DWI	DWI & T_2_w
1 Belue	4 Cochran	11 Johnson
2 Bischoff	9 Hu	18 Lee
3 Boschheidgen	10 Jeong	21 Oerther
5 Gassenmaier	14 Kaye	
6 Gassenmaier	20 Nishioka	
7 Gassenmaier	23 Pfaff	
8 Harder	29 Ueda	
12 Jung	30 Ursprung	
13 Jurka		
15 Kim		
16 Kim		
17 Kim		
19 Liu		
22 Park		
24 Riederer		
25 Sato		
26 Shen		
27 Shirashi		
28 Tong		
31 van Lohuizen		
32 Wang		
33 Zhu		

**Table A2:** Cont.

Study Design		
Prospective		Retrospective
2 Bischoff		1 Belue
3 Boschheidgen		4 Cochran
5 Gassenmaier		6 Gassenmaier
7 Gassenmaier		10 Jeong
8 Harder		11 Johnson
9 Hu		12 Jung
12 Jung		14 Kaye
13 Jurka		16 Kim
15 Kim		17 Kim
18 Lee		20 Nishioka
21 Oerther		22 Park
24 Riederer		23 Pfaff
25 Sato		27 Shirashi
26 Shen		28 Tong
32 Wang		29 Ueda
33 Zhu		30 Ursprung
		31 van Lohuizen

**Table A2:** Cont.

Deep Learning Type		
In-House Research	Vendor Research	Product
1 Belue	5 Gassenmaier	2 Bischoff
9 Hu	6 Gassenmaier	3 Boschheidgen
11 Johnson	7 Gassenmaier	4 Cochran
12 Jung	10 Jeong	8 Harder
14 Kaye	13 Jurka	15 Kim
19 Liu	17 Kim	18 Lee
23 Pfaff	27 Shirashi	20 Nishioka
31 van Lohuizen	28 Tong	21 Oerther
33 Zhu	30 Ursprung	22 Park
		24 Riederer
		25 Sato
		26 Shen
		29 Ueda
		32 Wang

**Table A2:** Cont.

Acceleration		
Simulated		Acquired
4 Cochran		2 Bischoff
11 Johnson		3 Boschheidgen
12 Jung		5 Gassenmaier
14 Kaye		6 Gassenmaier
23 Pfaff		7 Gassenmaier
30 Ursprung		8 Harder
31 van Lohuizen		10 Jeong
		12 Jung
		13 Jurka
		16 Kim
		17 Kim
		18 Lee
		21 Oerther
		22 Park
		26 Shen
		28 Tong
		33 Zhu

**Table A2:** Cont.

Clinical Validation			
PI-RADS	EPE Detection	Diagnostic Performance	Diagnostic AI Performance
2 Bischoff	15 Kim	10 Jeong	28 Tong
5 Gassenmaier	16 Kim	11 Johnson	31 van Lohuizen
6 Gassenmaier	22 Park	29 Ueda	
7 Gassenmaier			
8 Harder			
10 Jeong			
11 Johnson			
12 Jung			
16 Kim			
18 Lee			
21 Oerther			
26 Shen			
27 Shirashi			

Full references to the manuscript numbers and first authors can be found in Appendix B. Abbreviations: DWI: diffusion-weighted imaging, EPE: extraprostatic extension, T_2_w: T2-weighted.

## Appendix D. QUADAS-2 Assessment

High risk of bias was assessed in patient selection seven articles as described in the Section 3, with an unclear risk in five articles. Unclear risks of bias or applicability were identified for the index and reference test for 13 and 10 studies, respectively. The most common area of potential bias was the simulation of an accelerated acquisition as it might not produce identical results to truly accelerated acquisitions. There were no concerns about the flow and timing of the studies.

**Table A3:** QUADAS-2 analysis. of the risk of bias and applicability of the included studies.

#	Objective Image Quality Metrics		Index Test		Reference Test		Flow and Timing	
	**Risk of Bias**	**Applicability**	**Risk of Bias**	**Applicability**	**Risk of Bias**	**Applicability**	**Risk of Bias**	**Applicability**
1 Belue	☺	☺	☺	☺	☺	☺	☺	☺
2 Bischoff	☺	☺	☺	☺	☺	☺	☺	☺
3 Boschheidgen	😐︎	☺	☺	☺	☺	☺	☺	☺
4 Cochran	☺	☺	😐︎	😐︎	😐︎	☺	☺	☺
5 Gassenmaier	☺	☺	☺	☺	☺	☺	☺	☺
6 Gassenmaier	☺	☺	☺	☺	☺	☺	☺	☺
7 Gassenmaier	☺	☺	☺	☺	☺	☺	☺	☺
8 Harder	☺	☺	☺	☺	☺	☺	☺	☺
9 Hu	☹	☹	😐︎	☺	😐︎	☺	☺	☺
10 Jeong	☺	☺	☺	☺	☺	☺	☺	☺
11 Johnson	😐︎	😐︎	😐︎	😐︎	☺	☺	☺	☺
12 Jung	☺	☺	☺	☺	☺	☺	☺	☺
13 Jurka	☺	☺	☺	☺	☺	☺	☺	☺
14 Kaye	😐︎	😐︎	😐︎	😐︎	☺	☺	☺	☺
15 Kim	😐︎	☹	☺	☺	☺	☺	☺	☺
16 Kim	😐︎	☹	😐︎	😐︎	😐︎	☺	☺	☺
17 Kim	☺	☺	☹	☺	☹	☺	☺	☺
18 Lee	😐︎	☺	☺	☺	☺	☺	☺	☺
19 Liu	☺	☺	😐︎	😐︎	😐︎	😐︎	☺	☺
20 Nishioka	☹	☹	😐︎	😐︎	😐︎	😐︎	☺	☺
21 Oerther	☺	☺	☺	☺	😐︎	☺	☺	☺
22 Park	😐︎	☹	☺	☺	☺	☺	☺	☺
23 Pfaff	😐︎	😐︎	😐︎	☺	☺	☺	☺	☺
24 Riederer	☺	☺	😐︎	☺	😐︎	☺	☺	☺
25 Sato	☺	☹	☺	☺	☺	☺	☺	☺
26 Shen	☺	☺	☺	☺	☺	☺	☺	☺
27 Shirashi	☺	☺	😐︎	☺	😐︎	☺	☺	☺
28 Tong	☺	☺	☺	☺	☺	☺	☺	☺
29 Ueda	☹	☹	😐︎	☺	😐︎	☺	☺	☺
30 Ursprung	☺	☺	😐︎	😐︎	☺	☺	☺	☺
31 van Lohuizen	☺	☺	😐︎	😐︎	☺	☺	☺	☺
32 Wang	☺	☺	☺	☺	☺	😐︎	☺	☺
33 Zhu	☺	☺	☺	☺	☺	☺	☺	☺

☺ on green background: low risk of bias or applicability. 😐︎ on orange background: unclear risk of bias or applicability. ☹ on red background: high risk of bias or applicability.

## Appendix E. Statistical Methods

Supplementary statistical methods complementing the main methods: To quantify the heterogeneity between the studies in the meta-analysis, the I^2^ statistic was calculated. I^2^ is an estimate of the percentage of the variability in effect estimators that is attributable to heterogeneity between studies and cannot be explained by chance arising from sampling error. I^2^ values of 25% and less are usually considered to be low or unimportant, 25–50% moderate and values above 70% are considered high (Higgins JPT, Thompson SG (2002) Quantifying heterogeneity in a meta-analysis.). The computation of inter-study dispersion assumes that, effect sizes would follow a chi-squared distribution if variation was only due to random selection of participants and studies were methodologically identical (Higgins J, Thomas J, Chandler J, et al Cochrane Handbook for Systematic Reviews of Interventions version 6.0.).

A funnel plot was employed as a tool to explore the presence of publication bias among the studies included in the meta-analysis. The standardized mean difference of each study is plotted against its standard error, which is inversely related to sample size. An asymmetric distribution of studies on the funnel plot can indicate publication bias as a result of preferential reporting of significant studies. The dotted diagonal lines border the sector where 95% of all studies were assumed to lie (Sterne JAC, Egger M (2001) Funnel plots for detecting bias in meta-analysis: Guidelines on choice of axis).
